# Mating system, population growth, and management scenario for *Kalanchoe pinnata* in an invaded seasonally dry tropical forest

**DOI:** 10.1002/ece3.2219

**Published:** 2016-06-09

**Authors:** Salvador González de León, Ileana Herrera, Roger Guevara

**Affiliations:** ^1^ Instituto de Ecología A.C.Red de Biología EvolutivaCarretera Antigua a Coatepec 351El HayaXalapaVeracruzMéxico; ^2^ Facultad de Ciencias AgrariasUniversidad Agraria del EcuadorGuayaquilA.P. 01‐09‐1248Ecuador; ^3^ Centro de EcologíaInstituto Venezolano de Investigaciones CientíficasIVIC, A.P. 20632Caracas1020‐AVenezuela

**Keywords:** Biological invasion, discrete matrix model, *Kalanchoe*, management strategy, tropical forest

## Abstract

Ecological invasions are a major issue worldwide, where successful invasion depends on traits that facilitate dispersion, establishment, and population growth. The nonnative succulent plant *Kalanchoe pinnata*, reported as invasive in some countries, is widespread in remnants of seasonally dry tropical forest on a volcanic outcrop with high conservation value in east‐central Mexico where we assessed its mating system and demographic growth and identified management strategies. To understand its local mating system, we conducted hand‐pollination treatments, germination, and survival experiments. Based on the experimental data, we constructed a life‐stage population matrix, identified the key traits for population growth*,* weighted the contributions of vegetative and sexual reproduction, and evaluated management scenarios. Hand‐pollination treatments had slight effects on fruit and seed setting, as well as on germination. With natural pollination treatment, the successful germination of seeds from only 2/39 fruit suggests occasional effective natural cross‐pollination. The ratios of the metrics for self‐ and cross‐pollinated flowers suggest that *K. pinnata* is partially self‐compatible. Most of the pollinated flowers developed into fruit, but the seed germination and seedling survival rates were low. Thus, vegetative propagation and juvenile survival are the main drivers of population growth. Simulations of a virtual *K. pinnata* population suggest that an intense and sustained weeding campaign will reduce the population within at least 10 years. *Synthesis and applications*. The study population is partially self‐compatible, but sexual reproduction by *K. pinnata* is limited at the study site, and population growth is supported by vegetative propagation and juvenile survival. Demographic modeling provides key insights and realistic forecasts on invasion process and therefore is useful to design management strategies.

## Introduction

Biological invasions are among the most important threats to biodiversity throughout the world (Cronk and Fuller 1998; Hejda et al. 2009). Invasive plants can compete for space and resources with native plants, usually through rapid growth, high recruitment rates, and superior survival skills (Williamson and Fitter 1996; Van Kleunen, Weber & Fisher 2010), thereby altering the local population dynamics (Rejmánek and Richardson 1996; Strauss et al. 2006) as well as the community structure and composition (Vilà et al. 2011).

**Figure 1:**
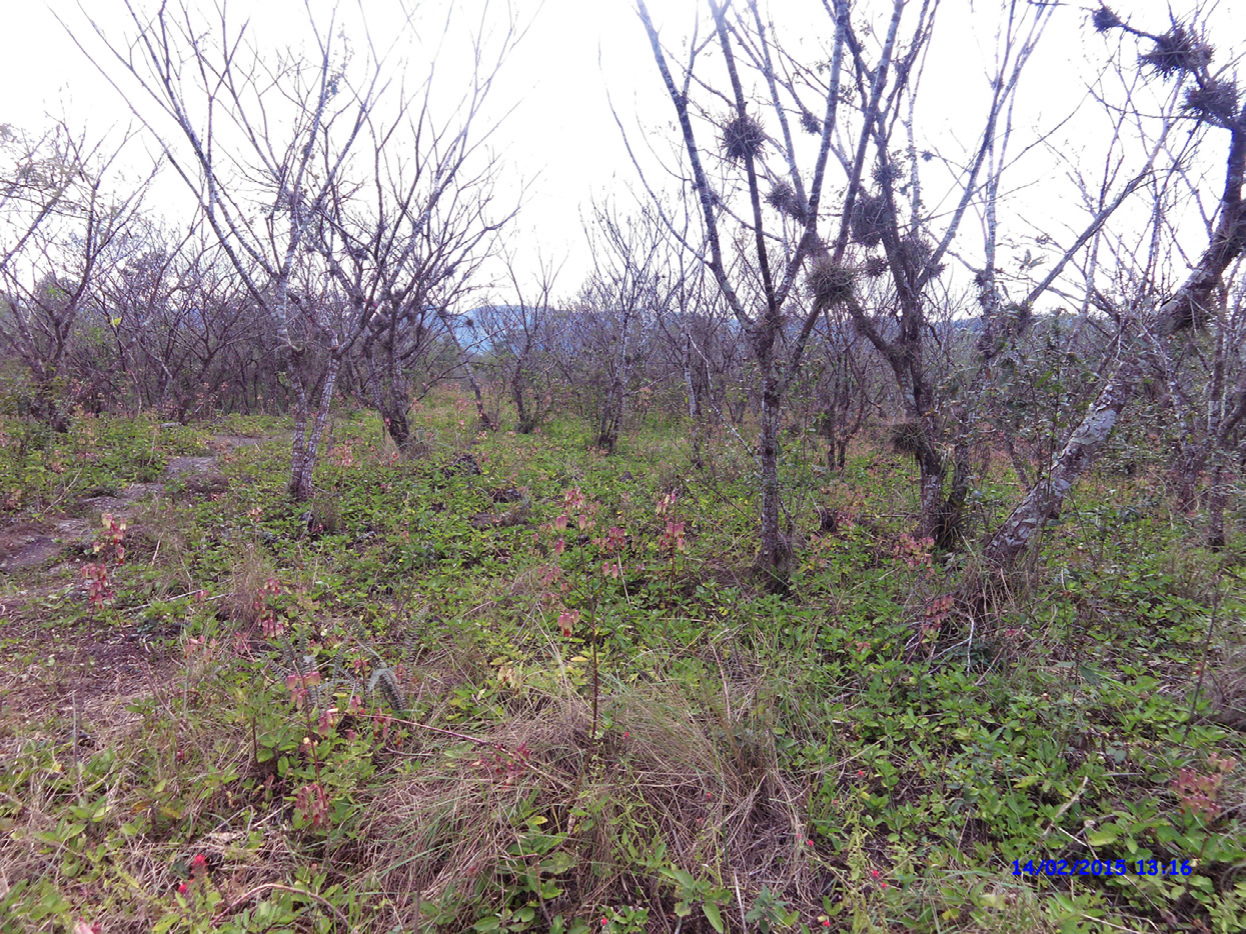
Understory view of the seasonal dry tropical forest established on volcanic substrate, central Veracruz, Mexico, with *Kalanchoe pinnata* invasion.

In the state of Veracruz in east‐central Mexico, the exotic plant *Kalanchoe pinnata* (Crassulaceae) has been reported over a wide altitudinal range and in different ecosystems (Sandoval and Martinez 1994), including seasonally dry tropical forest (SDTF). In particular, it appears to be most abundant in the SDTF found on volcanic rock (SDTFvr), where the presence of dense patches of *K. pinnata* ranging in area from tens to several hundreds of square meters (Fig. 1) is a specific concern to conservation biologists. The SDTFvr in central Veracruz grows on a 10,000‐year‐old consolidated lava flow, which is regarded as one of the main refuges for species from the Pleistocene glaciations (Arriaga et al. 2000). In floristic terms, this forest is one of the most diverse SDTFs on the Atlantic coast of Mexico and it harbors a high proportion of endemic species (Cházaro Basáñez 1992; Castillo‐Campos et al. 2007). Ongoing research in this region has shown that *K. pinnata* is a major driver of the ecological degradation of this community, including changes in the plant species composition and structure, as well as interfering with interactions such as herbivory and mycorrhizal colonization.

An approximation to understand the causes of invasions and facilitate the development of management strategies (Lott et al. 2003; Bonnett et al. 2014) relies on understanding the reproductive biology and demographic traits of invasive species. Here, in order to understand the reproductive biology of *K. pinnata*, we combined demographic structured population models with matrix perturbations methods (sensitivity and elasticity) to identify key life stages in the *K. pinnata* population (Zamora et al. 1989; Shea and Kelly 1998; De Kroon et al. 2000; Caswell 2001).

The genus *Kalanchoe* is native to Madagascar and southern Africa (Gehrig et al. 2001), but many species have been introduced worldwide for ornamental purposes because of their colorful and dense blooms (Baldwin 1938); in central Veracruz, formal records of *K. pinnata* date back to 1967 (XAL‐81942). Asexual propagation is a common characteristic of many *Kalanchoe* species and self‐compatibility has also been reported in some species of Crassulaceae (Jones et al. 2010), including the invasive *K. daigremontiana,* which is reported to be autogamous (Herrera and Nassar 2009), and *K. pinnata* (partially self‐compatible) in Venezuela (Ramírez & Nelson 1999). The combination of partially self‐compatible sexual and asexual reproductive traits that facilitate long‐ and short‐range dispersion respectively is an important determinant of invasiveness in nonnative plants (Baker 1955; Simberloff 2009). Without the need for allogamous fertilization, the colonization of new areas does not require pollinators and uniparental breeders are more likely to be invaders than self‐incompatible species (Allard et al. 1965; Baker 1974; Sutherland 2004). In the case of self‐incompatible perennials, invasiveness can be triggered by shifts in resource allocation that favor clonal growth, which reduces the flowering and seed production costs and increases vegetative propagation (Pannell and Barrett 1998). However, the self‐compatibility of invasive plants may vary in complex ways (Petanidou et al. 2012). In general, it is known that transitions from self‐compatibility to self‐incompatibility depend on the particular life history traits of a species (Igic et al. 2008). These shifts can be crucial for colonizing new areas, especially in semelparous and short‐lived perennial species with limited sexual reproduction and when pollinators might be a limiting factor (Montero‐Castaño et al. 2014). While this information is fragmentary or nonexisting for *K. pinnata*, it is of vital information to understand and manage invaded sites.

Overall, this study addressed the following questions. (1) What is the mating system of *K. pinnata* in the SDTFvr? (2) Which of the life stages of *K. pinnata* are the main drivers of demographic growth in the SDTFvr? (3) What are the relative contributions of sexual and vegetative reproduction to population growth by *K. pinnata* in the SDTFvr? (4) Can invasion of *K. pinnata* be managed with simple weeding efforts in the SDTFvr? Based on evidence, we predict that the invasive potential of *K. pinnata* in the SDTFvr is driven mainly by vegetative reproduction and it is supported by partial self‐compatibility.

## Materials and Methods

### Study site

The study was conducted in a 9‐km^2^ fragment of SDTFvr, which is the largest remnant of this vegetation type in the region. The study site is located 7 km east of the city of Xalapa, Veracruz, Mexico (19° 36′ 07.8″ N, 96°52′21.4″ W). Based on the Almolonga weather station (19° 35′ 17.9″ N, 96° 47′ 3.1″ W), the mean annual temperature and total annual precipitation are 22.5°C and 1023 mm, respectively [National Meteorological System (Mexico) 1951–2010]. The study site has two clearly defined seasons: the dry season (November–May) and the rainy season (June–October). The predominant vegetation in the study site is SDTF, where *Lysiloma acapulcensis* (Fabaceae), *Dodonaea viscosa* (Sapindaceae), and *Quercus germana* (Fagaceae) are the dominant canopy species, whereas *Cnidoscolus aconitifolius* (Euphorbiaceae), *Psychotria erythrocarpa* (Rubiaceae), and *Hamelia patens* (Rubiaceae) predominate in the shrub stratum, as well as hundreds of species of herbs, many of which are endemic to this particular site (Castillo‐Campos et al. 2005). The landscape includes sugarcane crops and small goat farms (50–100 animals/farm), which has favored anthropization of the SDTF. Despite this, remnants of the SDTFvr are unlikely to be fully transformed because the soils (consolidated lava spills from the late Pleistocene) are unsuitable for agricultural practices and intensive livestock farming (Negendak et al. 1985). Therefore, invasive species such as *K. pinnata, K. tubiflora,* the African orchid *Oeceoclades maculata* (personal observations), and the grasses *Cynodon dactylon*,* Echinochloa colona*, and *Dactyloctenium aegyptium* (Castillo‐Campos et al. 2007) are the major threats to the biodiversity of this region.

### *Kalanchoe pinnata* reproduction in the SDTFvr

*Kalanchoe pinnata* is a hermaphrodite perennial herb, which reaches sexual maturation after 2 years (Wadhi & Mohan Ram 1967). In the invaded area, it flowers profusely from mid‐November to March. The seeds are small (<1 mm in length), and they are readily dispersed by wind and rain or transported in animal fur (Hannan‐Jones and Playford 2002). In addition, it exhibits clonal regeneration from roots and, more importantly, from detached leaves. New clonal plantlets are potentially formed from all undifferentiated meristem in the marginal notches of the crenated leaves (Jaiswal and Sawhney 2008).

### Mating system, germination, and self‐incompatibility

In 2013, we marked and followed 242 flowers and recorded the time of maturation of sexual organs. During March in 2013 and 2014, 30 and 47, respectively, flowering *K. pinnata* plants were selected haphazardly from the study site. The selected plants were located at least 10 m from each other, and a plant was considered suitable for the experiment if the panicle had at least four floral buds. All of the floral buds were excluded from pollinators using a mesh bag (0.5 mm). As the buds matured, we applied one of four treatments to each plant, as follows. (1) Natural pollination (NP): The mesh bag was removed from a mature flower and exposed to pollinators. (2) Cross‐pollination (CP): A flower was hand‐pollinated with exogamous pollen using a blend of pollen grains from at least three donor plants. (3) Autopollination (AP): A flower was hand‐pollinated with pollen from the same plant. (4) Emasculation (Em): The anthers were removed before the flower was active. All of the experimental flowers were kept inside the mesh bags for 40 days, except for those that were naturally pollinated.

We conducted germination assays using the seeds from the pollination treatments. We used 3‐cm‐diameter polystyrene plates containing sterile sand, in which we sowed a known number of seeds (50–120) from each fruit. All of the trays were watered uniformly to keep the sand fully wet, and they were maintained at a local glasshouse facility for 55 days. The germinating seeds were recorded daily.

Indexes of self‐incompatibility (ISI) were calculated as the relative ratios between the metrics in the AP and CP treatments. This metric ranges from one (self‐compatibility) to zero (total self‐incompatibility), while values 0.2< ISI <1 indicate mostly self‐compatibility and ISI < 0.2 indicates mostly self‐incompatibility. The metrics used to estimate the ISI comprised the proportion of fruits formed relative to the number of pollinated flowers or fruit set (Zapata and Arroyo 1978), the ratio of mature seeds in each fruit relative to the number of ovules or seed set (Jaimes and Ramírez 1999), and the proportion of seeds that germinated. To perform comparisons among years and treatments, we performed analysis of variance (ANOVA) where the data were transformed in ranks with maximum rank possible assigned to tied values. This method is equivalent to nonparametric ANOVA (Conover and Iman 1981). Specific comparisons among years and treatments were performed as contrasts based on the linear predictor of the model (Warnes et al. 2015). The analyses were conducted in R (R Development Core Team 2008).

### Contributions of sexual and vegetative reproduction

To assess the contributions of sexual and vegetative reproduction to population growth, we constructed a simple time‐based matrix model (Leslie 1945; Lefkovitch 1965), which was parameterized using field and glasshouse experimental data (Eq. (1)). The model considers five life cycle stages: (1) seeds, (2) seedlings (sexual propagules), (3) plantlets (vegetative propagules), (4) juveniles, and (5) adults (individuals that reach the flowering stage). *A* is the population matrix, which represents individuals in stage *j* that survive, grow, or add (reproduction) to reach stage *i* in one time lap (1 year) (Table 4), and *n*_*t*_ is a vector of the number of individuals in each stage at a given time (*t*). It should be noted that the main diagonal in *A* represents the survival (*S*_ij_) of individuals in the same stage for a year. The *P*_*ij*_ values over the diagonal are related to fertility, which we refer to as *R*_ij_ (reproduction: sexual and vegetative), and the *P*_*ij*_ values below the diagonal are related to growth (*G*_ij_). To avoid unrealistic delays (Werner and Caswell 1977), the matrix was constructed by considering that seeds can germinate in the year they develop or they can become part of the seed bank.

(1) $$\begin{array}{cc} n_{t+1} & =An_{t};\\ A & =\left(\begin{array}{ccccc} S_{11} & 0 & 0 & 0 & R_{15}\\ G_{21} & S_{22} & 0 & 0 & R_{25}\\ 0 & 0 & S_{33} & R_{34} & R_{35}\\ 0 & G_{42} & G_{34} & S_{44} & 0\\ 0 & 0 & 0 & G_{54} & S_{55} \end{array}\right),n_{t}=\left(\begin{array}{c} n_{1}\\ n_{2}\\ n_{3}\\ n_{4}\\ n_{5} \end{array}\right) \end{array}$$

The transition matrix was parameterized according to the following considerations, the details of which are presented in Table S1. The viability of 1‐year‐old seeds (S_11_) was tested with tetrazolium by following standard procedures (International Seed Testing Association 1985). The proportion of seedlings that survived per year (S_22_) and the proportion of vegetative plantlets that survived per year (S_33_) were assessed under glasshouse conditions in pots containing vermiculite:soil (2:6) with regular watering. The *proportion of juveniles surviving per year (S*_*44*_) was assessed by following field‐tagged plants. *K. pinnata* is a semelparous species, so all plants that reached the adult stage (flowered) died in the model (*S*_*55*_) and we ignored the occasional resprouting from underground stolons (Hannan‐Jones and Playford 2002). The *proportion of seeds germinating from the seed bank per year (G*_*21*_) was estimated as the product of the ratio of seeds that did not germinate in a straightforward manner and S_11_. *The proportion of seedlings growing to the juvenile stage (G*_*42*_) was estimated by following 55‐day‐old seedlings transplanted to a vermiculite:soil mix (2:6) from August 2013 until August 2014. The *proportion of vegetative plantlets growing to the juvenile stage (G*_*43*_) was estimated by following vegetative plantlets (*S*_*33*_). For juvenile growth, the *proportion of juvenile plants growing into adults (G*_*54*_) was estimated by averaging two metrics based on field data. First, we followed tagged juvenile individuals from February 2013 to February 2014. Subsequently, in 2015, we surveyed twenty 1‐m^2^ plots with dense populations of *K. pinnata* by counting all of the juveniles that became adults (flowered). The fertility of adults or s*eeds produced per adult per year (R*_*15*_) was specified as the product of the mean number of fruit initiated per plant (*n* = 22), the number of seeds per fruit (*n* = 881), the probability that a fruit reached maturity (0.77), and the proportion of nongerminated seeds. The *average number of seeds that germinate in a year (R*_*25*_) was estimated as the germinated seed proportion multiplied by the number of seeds produced per inflorescence. The *average number of plantlets potentially produced per juvenile per year (R*_*34*_) and the a*verage of plantlets potentially produced per adult per year (R*_*35*_) were estimated as the number of dropped leaves (we followed tagged leaves of 60 juveniles and 20 adults plants in the field) and multiplying plantlets produced per leaf (based on 150 detached leaves from each juvenile and adult). Demographic analysis was performed, where eigenanalysis and perturbation analyses of sensitivity and elasticity were conducted using the popbio package in R (Stubben and Milligan 2007).

### Modeling projections and management scenarios

To obtain insights into the management of *K. pinnata*, we projected its demographic growth based on field data corresponding to dense patches of *K. pinnata*, where we assumed asymptotic steady‐state growth (Ezard et al. 2010). We computed a virtual population, which started from one adult and grew for 50 years. We introduced stochasticity by allowing each *P*_*ij*_ value in the transition matrix to vary randomly each time lap (Horvitz and Schemske 1995) according to a normal distribution around each observed *P*_*ij*_ value, where the standard deviation was equal to half of the *P*_*ij*_ value. The basis for these last assumptions was the empiric distribution of observed probabilities of juvenile growth. The model was specified with a carrying capacity of 17 juveniles per square meter based on the maximum observed field density, and at this point, it oscillated according to a random normal distribution with the mean and standard deviation observed in densely populated plots.

To validate the modeled projection, we compared the predicted individual numbers per stage with observations obtained from densely populated 1‐m^2^ field plots at the study site. Under field conditions, it is not possible to distinguish seedlings (sexual origin) and plantlets (asexual origin), so these metrics were added to perform the contrasts with the model's predictions. We used a simple mean comparison *t*‐test to compare the observed and simulated values.

We also modeled the management scenarios, including virtual efforts at *K. pinnata* weeding (between 0% and 100%). All of the analyses were computed in R (R Development Core Team 2008).

## Results

### Sexual reproduction and mating

**Figure 2:**
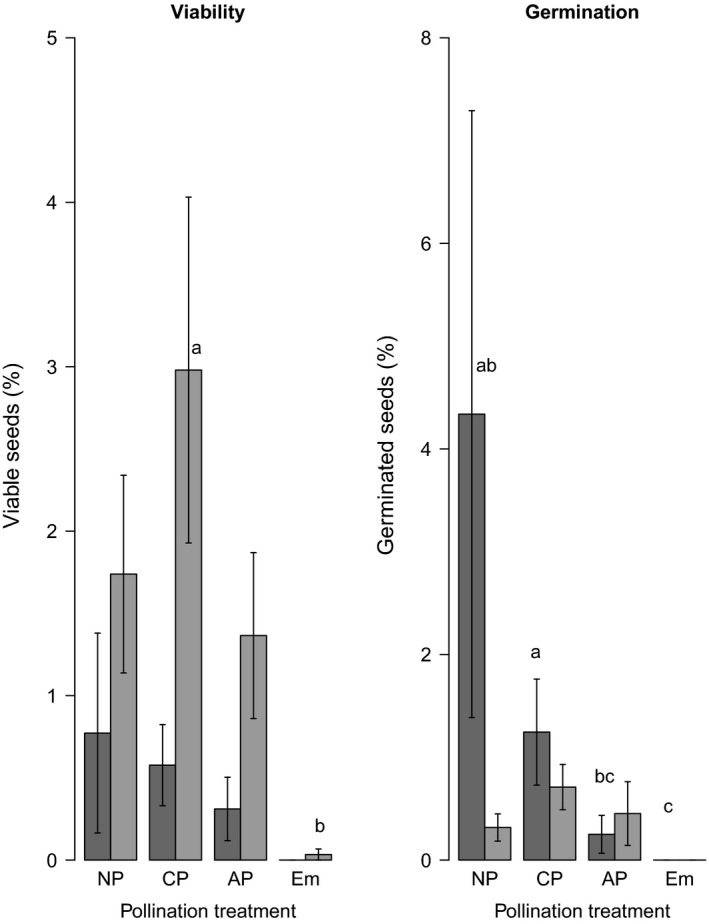
Percentage of seed viability and germination in 2013 (dark bars) and 2014 (clear bars) in pollination treatments: natural pollination (NP), cross‐pollination (CP), autogamous pollination (AP), and emasculation (Em).

*Kalanchoe pinnata* exhibited characteristics that suggest allogamy as the predominant breeding system. Almost 90% of the flowers were protandric, whereas in 10% of them sexual organs matured simultaneously with no herkogamy. Despite that, *K. pinnata* was mostly self‐compatible, and only viable seeds and germinated seeds suggested self‐incompatibility in 2013. The fructification, viability, and germination sets with different pollination treatments are presented in Table 1. ANOVA detected significant differences between the pollination treatments (Table 2). The overall viabilities with NP, CP, and AP were higher than that with Em, and the CP‐germinated seeds had higher viability than the Em seeds. In 2013, the viability did not differ between treatments, but more seeds germinated from the NP and CP treatments than Em, and more from CP than AP. In 2014, the viability with CP was higher than that with Em, and there were no differences in germination between treatments (Fig. 2). The NP treatment had the highest germination rate in 2013 (nearly 5%), but the overall germination rate was very low (<1%).

**Table 1:** Performance of *Kalanchoe pinnata* under different pollination treatments and self‐incompatibility index

	Natural pollination	Cross‐pollination	Autopollination	Emasculation	Index of self‐incompatibility^a^
Fruits/Flowers (2013)	(12/30)	(12/30)	(22/30)	(23/30)	1.4
Fruits/Flowers (2014)	(35/47)	(40/47)	(38/47)	(40/47)	0.95
Viable/Unviable Seeds(2013)	(15/922)	(46/1404)	3/1238	0/1211	**0.07**
Viable/Unviable Seeds (2014)	(38/1593)	(70/1739)	28/1676	1/1691	0.41
Germinated/NG seeds (2013^b^)	76/1541	42/2366	8/2523	2/2613	**0.18**
Germinated/NG seeds (2014)	16/5068	39/4925	33/4587	0/4041	0.9

NG = not germinated.

a ISI < 0.2 indicates self‐incompatibility (Bold numbers).

b In 2013, seeds were 1 year old.

**Table 2:** Summary of ANOVAs for viability and seed germination

	d.f.	Sum Sq.	Mean Sq.	*F* value	*P*
(a) Seed viability					
Pollination Treatment	3	2875	958.42	7.05	0.0001
Year	1	1047	1047	7.71	0.005
Pollination Treatment*Year	3	558	186	1.36	0.252
(b) Germination					
Pollination Treatment	3	1041	347	6.7109	0.0002
Year	1	56.3	56.33	1.0895	0.297
Pollination Treatment*Year	3	72.6	24.19	0.467	0.704

### Demography: matrix parameterization and modeling

The parameterization of the matrix is presented in Table 3 and details in Table S1. Eigenanalysis yielded a *λ *= 2.81, and the stable stage proportions were as follows: seeds = 0.974, seedlings = 0.019, plantlets = 0.0044, juveniles = 0.002, and adults = 0.0002. The highest sensitivities to *λ* were associated with *G*_*21*_ (seed to seedling) = 6.16, *G*_*42*_ (seedling to juvenile) = 4.02, and *G*_*54*_ (juvenile to adult) = 1.857 (Table 4), while the highest elasticities of *λ* were related to *G*_*43*_ (plantlet growth) = 0.253, *R*_*34*_ (asexual reproduction of juveniles) = 0.2, *G*_*54*_ (juvenile to adult transition) = 0.171, and *G*_*42*_ (seedling growth) = 0.119 (Table 4).

**Table 3:** Life‐stage transition matrix of *Kalanchoe pinnata* based on simple time‐stage population matrix models (Leslie 1945; Lefkovitch 1965), parameterized based on both field and glasshouse experiments and observations

Life Stage	Seed	Seedling	Plantlet	Juvenile	Adult
Seed	0.016				14924
Seedling	0.02	0.06			205
Plantlet			0.05	4.83	13.7
Juvenile		0.083	0.78	0.28	
Adult				0.26	

**Table 4:** Sensitivity/elasticity matrix of *Kalanchoe pinnata* in a seasonally dry tropical forest growing on volcanic rock in central Veracruz

	Seed	Seedling	Plantlet	Juvenile	Adult
Seed	0.035/<0.001				0/0.035
Seedling	**6.16**/0.035	0.12/0.003			0.001/0.084
Plantlet			0.26/0.005	0.12/**0.201**	0.011/0.052
Juvenile		**4.02**/**0.119**	0.91/**0.253**	0.41/0.042	
Adult				**1.86**/**0.172**	

The model projections showed that the *K. pinnata* population achieved an asymptotic state at approximately 5 years after its introduction and the simulated mean numbers of adults, juveniles, and plantlets did not differ from those observed in densely populated patches of *K. pinnata* (*t* < 0.01, *P* = 0.99; *t* = 0.2, *P* = 0.82; *t* = 0.2, *P* = 0.82, respectively).

**Figure 3:**
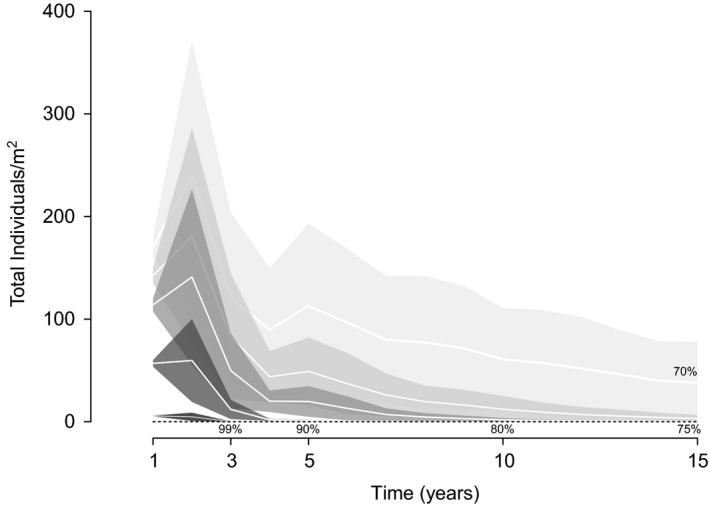
Effects of weeding scenarios efforts (70%, 75%, 80%, 90%, 99%) on the population growth of *Kalanchoe pinnata*. White lines correspond to the mean, and polygons are the standard deviation of 1000 simulations.

The simulated management actions indicated that annual weeding efforts at sensible and elastic life stages (seedlings, plantlets, juveniles, and adults) would decrease the population growth if 75% of the total individuals are removed, but removing >80% would reduce population to zero in at least 10 years (Fig. 3).

## Discussion

Our results showed that the *K. pinnata* is partially self‐compatible, but sexual reproduction has a minor contribution in the demography as the seed germination rate is very low. Population expansion and permanence are supported by propagule growth and survival (Lockwood et al. 2005; Simberloff 2009), which are probably driven by diffusion‐based expansion (Sakai et al. 2001), with few events involving long‐distance colonization. The absence of effective pollinators (Parker 1997) may be a relevant factor that limits sexual reproduction by *K. pinnata* in the SDTFvr, but experimental pollination treatments indicated the extensive nonviability of sexual offspring. According to the tetrazolium test, <4% of seeds were viable, and thus, the overall seed germination rate was low (<1%). The ratio of germinated seeds in the CP treatment relative to the AP treatment indicated that the self‐incompatibility of *K. pinnata* varied greatly over time at the study site, where it mainly changed from self‐incompatible (0.07) in 2013 to partially self‐compatible (0.4) in 2014. The fitness consequences of selfing (i.e., depression by endogamy), the likelihood of true outcrossing, and mate availability may be underlying factors that explain the observed variability in ISI (Sakai and Weller 1999; Barrett 2002; Willi 2009). Clonal propagules supported population growth at the study site, so it is likely that unintentionally the pollen of clonal offspring was used in the CP treatments even when the donors were selected at a considerable distance from the focal plants. However, our results suggest that occasional true outcrossing may have occurred because in the NP treatment during 2013, there was an unusually high contribution from seed germination by two individuals (two fruit) with seed germination ratios of 28% and 32%, which was 24 times higher compared with the overall average (1.25%) by all of the other naturally pollinated fruits. Therefore, dispersed seeds may occasionally facilitate the colonization of distant areas in the invaded landscape, although the success of the sexual propagules is low; the survival of seedlings under controlled conditions was less than 5% after 1 year.

It is known that native or naturalized pollinators may visit alien plant species (Vilà et al. 2009). At the study site, we observed that bees (*Apis*) were visitors to *K. pinnata* flowers, and thus, they may occasionally transfer exogenous pollen. Despite all of the limitations observed in the sexual reproduction of *K. pinnata*, the ISI suggest that the population in the SDTFvr in central Veracruz is at least partially self‐compatible, which agrees with the study conducted by Jaimes and Ramírez (1999) in a deciduous forest in Venezuela. However, the study population of *K. pinnata* exhibited characteristics that suggested allogamy is the predominant breeding system (90% of protandric flowers). The observed polymorphic sexual expression may be explained by fast genetic processes or rapid evolution (Lee 2002; Buswell et al. 2011). Fast genetic selection may occur in partially self‐incompatible species, where rapidly evolving adaptations can facilitate self‐compatibility. In addition, some species in *Kalanchoe* are known to be polyploid (Baldwin 1938), a characteristic that can break self‐incompatibility mechanisms and provide opportunities for the evolution of self‐fertilization (Chawla et al. 1997). The low frequency of nonherkogamous plants in the SDTFvr may indicate the early stages of a microevolutionary shift in the breeding system of *K. pinnata* in a similar manner to the many self‐incompatible to self‐compatible transitions observed in other angiosperms (Barrett 2002; Igic et al. 2004; Mast et al. 2006; Ferrer and Good‐Avila 2007). Further investigations and constant monitoring of the population of *K. pinnata* in the SDTFvr are needed to confirm these changes, which may include an invasion meltdown involving *K. pinnata* and bees (Simberloff and Von Holle 1999), and a case of rapid evolution as the invasion process matures (Lee 2002; Maron et al. 2004).

The population growth of *K. pinnata* is mostly supported by vegetative reproduction, which is a trait shared with other invasive *Kalanchoe* species such as *K. daigremontiana* (Herrera et al. 2012). In addition, changes in life history traits such as seedling survival and juvenile growth can severely affect the population growth rate, whereas an increase in seed production is irrelevant. Increased germination success would trigger more frequent long‐range dispersal (Baker 1955), which highlights the need to closely monitor the seed germination rates in the population.

The demographic contribution of juveniles is directly related to the capacity to produce clonal plantlets from detached leaves (Garcês et al. 2007). Some physiological advantages that contribute to the population growth are water economy and plasticity, traits well known in the CAM plants (Lüttge et al. 1991; Winter and Smith 1996) and particularly in many *Kalanchoe* species what may favor their invasive behavior (Guerra‐García et al. 2014). Clonal reproduction and plasticity are strategies employed by *K. pinnata* under natural conditions in its native distribution, but in the invaded area, the evergreen *K. pinnata* seems to be more effective at colonizing free spaces than native deciduous plants (Colautti et al. 2004). Recent evidence (to be published elsewhere) shows reductions in the plant community of the SDTFvr and changes in the soil biogeochemistry (cf. Ehrenfeld 2003; Vilà et al. 2011) associated with *K. pinnata* in the study site.

Population models provide a good approximation to identify promising approaches to ecological management. The implemented model suggested that after an adult plant is established, it takes less than 10 years to populate one square meter. Despite its simplicity and limitations, the model was a fair representation of the actual population, as modeled estimates did not differ significantly from those observed in the study site, and it was accurate although the simulation only represented stochastic population growth ignoring diffusive expansion, density‐dependent interactions [inter‐ and intra‐allelochemical interactions (Bär, Pfeifer & Dettner 1997)], and changes in sensible or elastic life traits associated with population structure or transient dynamics (Caswell 2001; Ezard et al. 2010).

Population models provide a good approximation to identify promising approaches to ecological management, but their practical implementation is often not simple. Choosing between sensitivities and elasticities to implement effective control campaigns is not intuitive to managers. The simulations predicted that the population would decline if annual weeding efforts of ≥80% are sustained for at least 10 years, but only if the all stages highlighted by elasticity and sensibility analysis are removed (Fig. S1). In the ground even for the relatively small area of the study site, the level of weeding needed is of paramount dimension. Thus, a solution seems unlikely if it is motivated solely by conservation concerns. The key to population management may be based on containment (Fletcher et al. 2015) and finding innovative applications of *K. pinnata*. For example, it is known that *K. pinnata* is rich in pharmacological components (Pattewar 2012), so if a local market is created for the harvested plants, locals will be likely to be an effective control agent of the population.

In conclusion, sexual reproduction by *K. pinnata* was limited in the SDTFvr, even in manually cross‐pollinated flowers; the population growth is supported by the growth of seedlings and juveniles and the asexual propagation of the juveniles. Juveniles support the population directly by reaching the inflorescence stage and indirectly by contributing new sexual and asexual propagules. Moreover, the predominant vegetative reproduction promotes the adaptability of the population. *K. pinnata* is an invasive plant in the SDTFvr (cf. Richardson et al. 2000), and management actions should be considered with yearly weeding efforts to slow down the invasion by *K. pinnata* in the SDTFvr.

## Conflict of Interest

None declared.

## Supporting information


**Figure S1.** Management on simulated populations of *Kalanchoe pinnata*, when: elastic (a), sensible (b) or both (c) life traits are removed annually at a rate of 80%.

**Table S1.** Summary of vital rates estimation; *P*_ij_ values were estimated for 1 year discrete transitions.
**Table S2.** Summary of contrasts for viability and germination of seeds between pollination treatments applied in *Kalanchoe pinnata* based on 234 degrees of freedom.

## References

[ece32219-bib-0001] Allard, R. W. , H. G. Baker , and G. L. Stebbins . 1965 Genetic systems associated with colonizing ability in predominantly self‐pollinated species. The genetics of colonizing species, 49–75.

[ece32219-bib-0002] Arriaga, L. , J. M. Espinoza , C. Aguilar , E. Martínez , L. Gómez , and E. Loa (coordinadores). 2000 Regiones terrestres prioritarias de México. Comisión Nacional para el Conocimiento y uso de la Biodiversidad, México.

[ece32219-bib-0003] Baker, H. G. 1955 Self‐compatibility and establishment after ‘long‐distance’ dispersal. Evolution 9:347–349.

[ece32219-bib-0004] Baker, H. G. 1974 The evolution of weeds. Annu. Rev. Ecol. Syst. 5:1–24.

[ece32219-bib-0005] Baldwin, J. T. Jr . 1938 *Kalanchoe*: the genus and its chromosomes. Am. J. Bot. 25:572–579.

[ece32219-bib-0100] Bär, W. , P. Pfeifer , and K. Dettner . 1997 Intra‐and interspecific allelochemical effects in three *Kalanchoe*‐species (Crassulaceae). Zeitschrift für Naturforschung C 52:441–449.

[ece32219-bib-0006] Barrett, S. C. 2002 The evolution of plant sexual diversity. Nat. Rev. Genet. 3:274–284. 11967552 10.1038/nrg776

[ece32219-bib-0007] Bonnett, G. , J. Kushner , and K. Saltonstall . 2014 The reproductive biology of Saccharum spontaneum L.: implications for management of this invasive weed in Panama. NeoBiota 20:61–79.

[ece32219-bib-0008] Buswell, J. M. , A. T. Moles , and S. Hartley . 2011 Is rapid evolution common in introduced plant species? J. Ecol. 99:214–224.

[ece32219-bib-0009] Castillo‐Campos, G. , M. E. M. Abreo , P. D. Aranda , and J. A. Z. Hurtado . 2005 Contribución al conocimiento del endemismo de la flora vascular en Veracruz, México. Acta Bot. Mex. 73:19–57.

[ece32219-bib-0010] Castillo‐Campos, G. , P. Dávila‐Aranda , and J. A. Zavala‐Hurtado . 2007 La selva baja caducifolia en una corriente de lava volcánica en el centro de Veracruz: lista florística de la flora vascular. Boletín de la Sociedad Botánica de México 80:77–104.

[ece32219-bib-0011] Caswell, H. 2001 Matrix population models, Chapter 9. John Wiley & Sons, Ltd, Sinauer Associates, Inc. Publishers, Sunderland, MA, USA.

[ece32219-bib-0013] Chawla, B. , R. Bernatzky , W. Liang , and M. Marcotrigiano . 1997 Breakdown of self‐incompatibility in tetraploid Lycopersicon peruvianum: inheritance and expression of S‐related proteins. Theor. Appl. Genet. 95:992–996.

[ece32219-bib-0014] Cházaro Basáñez, M. D. J. 1992 Exploraciones botánicas en Veracruz y estados circunvecinos I. Pisos altitudinales de vegetación en el centro de Veracruz y zonas limítrofes con Puebla.

[ece32219-bib-0015] Colautti, R. I. , A. Ricciardi , I. A. Grigorovich , and H. J. MacIsaac . 2004 Is invasion success explained by the enemy release hypothesis? Ecol. Lett. 7:721–733.

[ece32219-bib-0016] Conover, W. J. , and R. L. Iman . 1981 Rank transformations as a bridge between parametric and nonparametric statistics. Am. Stat. 35:124–129.

[ece32219-bib-0017] Cronk, Q. C. B. , and J. L. Fuller . 1998 Plant invaders: the threat to natural ecosystems. Biodivers. Conserv. 7:267–269.

[ece32219-bib-0018] De Kroon, H. , J. Van Groenendael , and J. Ehrlén . 2000 Elasticities: a review of methods and model limitations. Ecology 81:607–618.

[ece32219-bib-0019] Ehrenfeld, J. G. 2003 Effects of exotic plant invasions on soil nutrient cycling processes. Ecosystems 6:503–523.

[ece32219-bib-0020] Ezard, T. H. , J. M. Bullock , H. J. Dalgleish , A. Millon , F. Pelletier , A. Ozgul , et al. 2010 Matrix models for a changeable world: the importance of transient dynamics in population management. J. Appl. Ecol. 47:515–523.

[ece32219-bib-0021] Ferrer, M. M. , and S. V. Good‐Avila . 2007 Macrophylogenetic analyses of the gain and loss of self‐incompatibility in the Asteraceae. New Phytol. 173:401–414. 17204086 10.1111/j.1469-8137.2006.01905.x

[ece32219-bib-0022] Fletcher, C. S. , D. A. Westcott , H. T. Murphy , A. C. Grice , and J. R. Clarkson . 2015 Managing breaches of containment and eradication of invasive plant populations. J. Appl. Ecol. 52:59–68. 25678718 10.1111/1365-2664.12361 PMC4312900

[ece32219-bib-0023] Garcês, H. M. , C. E. Champagne , B. T. Townsley , S. Park , R. Malhó , M. C. Pedroso , et al. 2007 Evolution of asexual reproduction in leaves of the genus *Kalanchoe* . Proc. Natl Acad. Sci. 104:15578–15583. 17893341 10.1073/pnas.0704105104 PMC2000513

[ece32219-bib-0024] Gehrig, H. , O. Gaußmann , H. Marx , D. Schwarzott , and M. Kluge . 2001 Molecular phylogeny of the genus *Kalanchoe* (Crassulaceae) inferred from nucleotide sequences of the ITS‐1 and ITS‐2 regions. Plant Sci. 160:827–835. 11297779 10.1016/s0168-9452(00)00447-7

[ece32219-bib-0025] Guerra‐García, A. , J. Golubov , and M. C. Mandujano . 2014 Invasion of *Kalanchoe* by clonal spread. Biol. Invasions 17:1615–1622.

[ece32219-bib-0027] Hannan‐Jones, M. A. , and J. Playford . 2002 The biology of Australian weeds 40. Bryophyllum Salisb. species. Plant Prot. Q. 17:42–58.

[ece32219-bib-0028] Hejda, M. , P. Pyšek , and V. Jarošík . 2009 Impact of invasive plants on the species richness, diversity and composition of invaded communities. J. Ecol. 97:393–403.

[ece32219-bib-0029] Herrera, I. , and J. M. Nassar . 2009 Reproductive and recruitment traits as indicators of the invasive potential of *Kalanchoe daigremontiana* (Crassulaceae) and Stapelia gigantea (Apocynaceae) in a Neotropical arid zone. J. Arid Environ. 73:978–986.

[ece32219-bib-0030] Herrera, I. , M. J. Hernandez , M. Lampo , and J. M. Nassar . 2012 Plantlet recruitment is the key demographic transition in invasion by *Kalanchoe daigremontiana* . Popul. Ecol. 54:225–237.

[ece32219-bib-0031] Horvitz, C. C. , and D. W. Schemske . 1995 Spatiotemporal variation in demographic transitions of a tropical understory herb: projection matrix analysis. Ecol. Monogr. 65:155–192.

[ece32219-bib-0033] Igic, B. , L. Bohs , and J. R. Kohn . 2004 Historical inferences from the self‐incompatibility locus. New Phytol. 161:97–105.

[ece32219-bib-0034] Igic, B. , R. Lande , and J. R. Kohn . 2008 Loss of self‐incompatibility and its evolutionary consequences. Int. J. Plant Sci. 169:93–104.

[ece32219-bib-0035] International Seed Testing Association . 1985 Seed testing handbook. I.S.T.A., Zurich.

[ece32219-bib-0036] Jaimes, I. , and N. Ramírez . 1999 Breeding systems in a secondary deciduous forest in Venezuela: the importance of life form, habitat, and pollination specificity. Plant Syst. Evol. 215:23–36.

[ece32219-bib-0037] Jaiswal, S. , and S. Sawhney . 2008 Thidiazuron‐induced hypertrophic growth from foliar disks of *Kalanchoe pinnata* leads to visualization of a bioactive auxin gradient across the leaf plane. In Vitro Cell. Dev. Biol. Plant 44:65–68.

[ece32219-bib-0038] Jones, C. E. , F. M. Shropshire , R. L. Allen , and Y. C. Atallah . 2010 Pollination and reproduction in natural and mitigation populations of the many‐stemmed Dudleya, Dudleya multicaulis (Crassulaceae). Madroño 57:42–53.

[ece32219-bib-0039] Lee, C. E. 2002 Evolutionary genetics of invasive species. Trends Ecol. Evol. 17:386–391.

[ece32219-bib-0040] Lefkovitch, L. P. 1965 An extension of the use of matrices in population mathematics. Biometrics 21:1–18.

[ece32219-bib-0041] Leslie, P. H. 1945 On the use of matrices in certain population mathematics. Biometrika 33:183–212. 21006835 10.1093/biomet/33.3.183

[ece32219-bib-0042] Lockwood, J. L. , P. Cassey , and T. Blackburn . 2005 The role of propagule pressure in explaining species invasions. Trends Ecol. Evol. 20:223–228. 16701373 10.1016/j.tree.2005.02.004

[ece32219-bib-0043] Lott, M. S. , J. C. Volin , R. W. Pemberton , and D. F. Austin . 2003 The reproductive biology of the invasive ferns Lygodium microphyllum and L. japonicum (Schizaeaceae): implications for invasive potential. Am. J. Bot. 90:1144–1152. 21659214 10.3732/ajb.90.8.1144

[ece32219-bib-0044] Lüttge, U. , E. Ball , M. Fetene , and E. Medina . 1991 Flexibility of crassulacean acid metabolism in Kalanchoë pinnata (Lam.) Pers. I. Response to irradiance and supply of nitrogen and water. J. Plant Physiol. 137:259–267.

[ece32219-bib-0045] Maron, J. L. , M. Vilà , R. Bommarco , S. Elmendorf , and P. Beardsley . 2004 Rapid evolution of an invasive plant. Ecol. Monogr. 74:261–280.

[ece32219-bib-0046] Mast, A. R. , S. Kelso , and E. Conti . 2006 Are any primroses (Primula) primitively monomorphic? New Phytol. 171:605–616. 16866962 10.1111/j.1469-8137.2006.01700.x

[ece32219-bib-0047] Montero‐Castaño, A. , M. Vilà , and F. J. Ortiz‐Sánchez . 2014 Pollination ecology of a plant in its native and introduced areas. Acta Oecol. 56:1–9.

[ece32219-bib-0048] Negendak, J. F. , R. Emmermann , R. Krawczyk , F. Mooser , H. Tobschall , and D. Werle . 1985 Geological and geochemical investigations on the eastern trans mexican volcanic belt. Geofisica Internacional 24:477–575.

[ece32219-bib-0049] Pannell, J. R. , and S. C. Barrett . 1998 Baker's law revisited: reproductive assurance in a metapopulation. Evolution 52:657–668. 10.1111/j.1558-5646.1998.tb03691.x 28565251

[ece32219-bib-0050] Parker, I. M. 1997 Pollinator limitation of Cytisus scoparius (Scotch broom), an invasive exotic shrub. Ecology 78:1457–1470.

[ece32219-bib-0052] Pattewar, S. V. 2012 *Kalanchoe pinnata*: Phytochemical and pharmacological profile. Int. J. Phytopharmacy 2:1–8.

[ece32219-bib-0053] Petanidou, T. , R. C. Godfree , D. S. Song , A. Kantsa , Y. L. Dupont , and N. M. Waser . 2012 Self‐compatibility and plant invasiveness: Comparing species in native and invasive ranges. Perspect. Plant Ecol. Evol. Syst. 14:3–12.

[ece32219-bib-0054] R Development Core Team . 2008 R: a language and environment for statistical computing. R Foundation for Statistical Computing, Vienna, Austria ISBN 3‐900051‐07‐0, URL http://www.R-project.org.

[ece32219-bib-0055] Rejmánek, M. , and D. M. Richardson . 1996 What attributes make some plant species more invasive? Ecology 77:1655–1661.

[ece32219-bib-0056] Richardson, D. M. , P. Pyšek , M. Rejmánek , M. G. Barbour , F. D. Panetta , and C. J. West . 2000 Naturalization and invasion of alien plants: concepts and definitions. Divers. Distrib. 6:93–107.

[ece32219-bib-0058] Sakai, A. K. , and S. G. Weller . 1999 Gender and sexual dimorphism in flowering plants: a review of terminology, biogeographic patterns, ecological correlates, and phylogenetic approaches Pp. 1–31. *in* Geber M.A., Dowson T.E. and Delph L.F., eds. Gender and sexual dimorphism in flowering plants. Springer, Berlin, Heidelberg.

[ece32219-bib-0059] Sakai, A. K. , F. W. Allendorf , J. S. Holt , D. M. Lodge , J. Molofsky , K. A. With , et al. 2001 The population biology of invasive specie. Annu. Rev. Ecol. Syst. 32:305–332.

[ece32219-bib-0060] Sandoval, M. C. , and J. L. Martinez . 1994 El uso de Kalanchoe pinnata (Lam.) Pers. En el estado de Veracruz. Facultad de Biologia Universidad Veracruzana, Instituto de Ecologia A.C., Ver., Mexico.

[ece32219-bib-0061] Shea, K. , and D. Kelly . 1998 Estimating biocontrol agent impact with matrix models: Carduus nutans in New Zealand. Ecol. Appl. 8:824–832.

[ece32219-bib-0062] Simberloff, D. 2009 The role of propagule pressure in biological invasions. Annu. Rev. Ecol. Evol. Syst. 40:81–102.

[ece32219-bib-0063] Simberloff, D. , and B. Von Holle . 1999 Positive interactions of nonindigenous species: invasional meltdown? Biol. Invasions 1:21–32.

[ece32219-bib-0064] Strauss, S. Y. , C. O. Webb , and N. Salamin . 2006 Exotic taxa less related to native species are more invasive. Proc. Natl Acad. Sci. 103:5841–5845. 16581902 10.1073/pnas.0508073103 PMC1421337

[ece32219-bib-0065] Stubben, C. , and B. Milligan . 2007 Estimating and analyzing demographic models using the popbio package in R. J. Stat. Softw. 22:1–23.

[ece32219-bib-0066] Sutherland, S. 2004 What makes a weed a weed: life history traits of native and exotic plants in the USA. Oecologia 141:24–39. 15300483 10.1007/s00442-004-1628-x

[ece32219-bib-0102] Van Kleunen, M. , E. Weber , and M. Fischer . 2010 A meta‐analysis of trait differences between invasive and non‐invasive plant species. Ecology Letters 13:235–245. 20002494 10.1111/j.1461-0248.2009.01418.x

[ece32219-bib-0067] Vilà, M. , I. Bartomeus , A. C. Dietzsch , T. Petanidou , I. Steffan‐Dewenter , J. C. Stout , et al. 2009 Invasive plant integration into native plant–pollinator networks across Europe. Proc. R. Soc. Lond. B Biol. Sci. 276:3887–3893. 10.1098/rspb.2009.1076 PMC2817287 19692403

[ece32219-bib-0068] Vilà, M. , J. L. Espinar , M. Hejda , P. E. Hulme , V. Jarošík , J. L. Maron , et al. 2011 Ecological impacts of invasive alien plants: a meta‐analysis of their effects on species, communities and ecosystems. Ecol. Lett., 14:702–708. 21592274 10.1111/j.1461-0248.2011.01628.x

[ece32219-bib-0101] Wadhi, M. , and H. M. Ram . 1967 Shortening the juvenile phase for flowering in *Kalanchoe pinnata* Pers. Planta 73:28–36. 24554366 10.1007/BF00419838

[ece32219-bib-0069] Warnes, G. R. , B. Bolker , T. Lumley , and R. C. Johnson . 2015 Gmodels: various R programming tools for model fitting. SAIC‐Frederick and Inc, Funded by the Intramural Research Program, of the NIH, National Cancer Institute, Center for Cancer Research under NCI Contract NO1‐CO‐12400.

[ece32219-bib-0070] Werner, P. A. , and H. Caswell . 1977 Population growth rates and age versus stage‐distribution models for teasel (Dipsacus sylvestris Huds.). Ecology 58:1103–1111.

[ece32219-bib-0071] Willi, Y. 2009 Evolution towards self‐compatibility when mates are limited. J. Evol. Biol. 22:1967–1973. 19702891 10.1111/j.1420-9101.2009.01806.x

[ece32219-bib-0072] Williamson, M. , and A. Fitter . 1996 The varying success of invaders. Ecology 77:1661–1666.

[ece32219-bib-0073] Winter, K. , and J. A. C. Smith . 1996 An introduction to crassulacean acid metabolism. Biochemical principles and ecological diversity Pp. 1–13 *in* Lange O.L. and Mooney H.A., eds. Crassulacean acid metabolism. Springer, Berlin, Heidelberg.

[ece32219-bib-0074] Zamora, D. L. , D. C. Thill , and R. E. Eplee . 1989 An eradication plan for plant invasions. Weed Technol. 3:2–12.

[ece32219-bib-0075] Zapata, T. R. , and M. T. K. Arroyo . 1978 Plant reproductive ecology of a secondary deciduous tropical forest in Venezuela. Biotropica 10:221–230.

