# Supplementary material for: Mating system, population growth, and management scenario for Kalanchoe pinnata in an invaded seasonally dry tropical forest
Source: Ecol Evol. 2016 Jun 9;6(13):4541–50. doi: 10.1002/ece3.2219 (PMC4931000; doi:10.1002/ece3.2219)
Supplement: Supplementary file 1 — Figure S1. Management on simulated populations of Kalanchoe pinnata, when: elastic (a), sensible (b) or both (c) life traits are removed annually at a rate of 80%. [file ECE3-6-4541-s001.pdf]

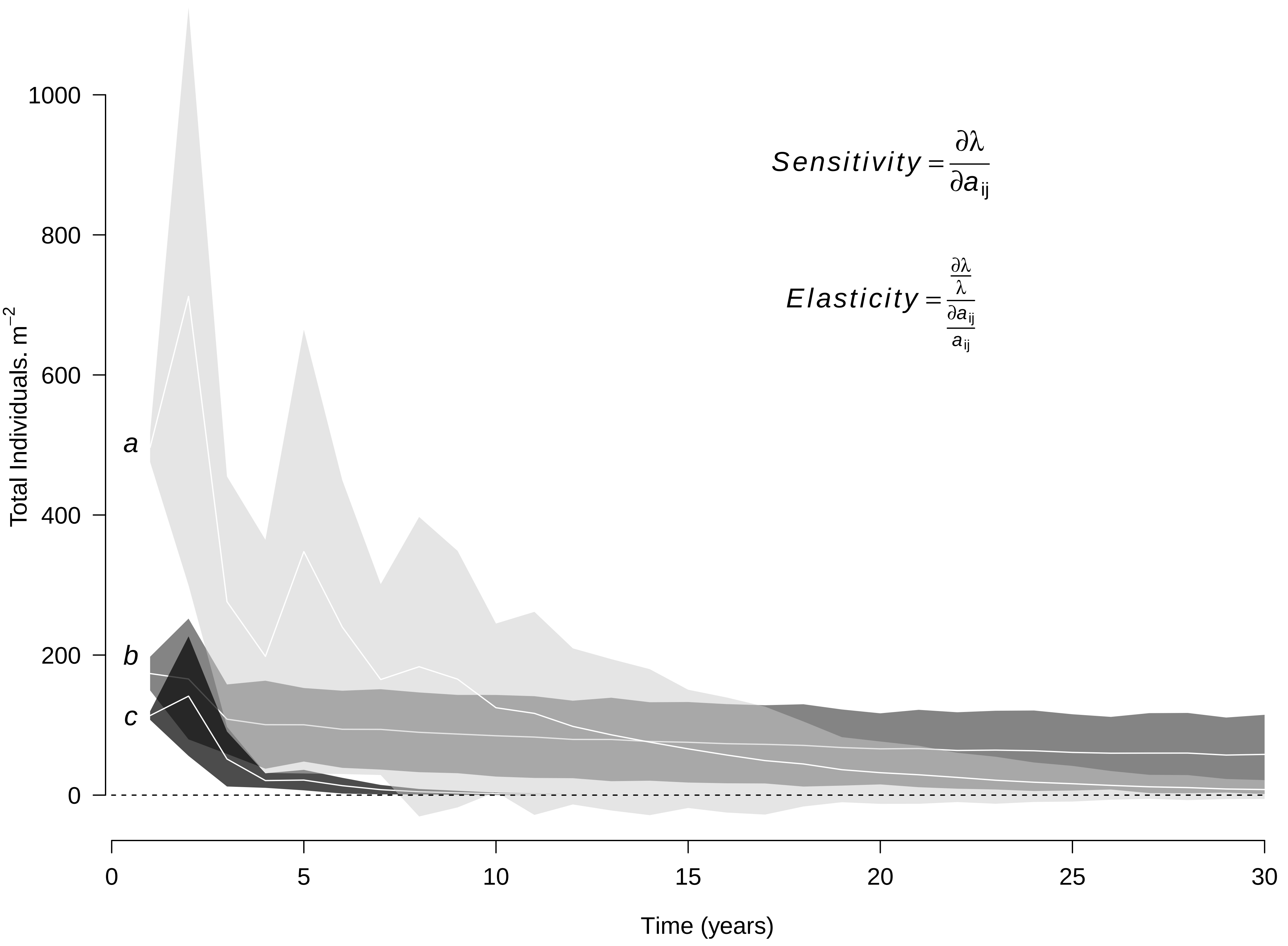

1000
800
600
400
200
0
Total Individuals. m⁻²
0
5
10
15
20
25
30
Time (years)
a
b
c
$$Sensitivity = \frac{\partial\lambda}{\partial a_{ij}}$$
$$Elasticity = \frac{\frac{\partial\lambda}{\lambda}}{\frac{\partial a_{ij}}{a_{ij}}}$$
