# Supplementary material for: Mating system, population growth, and management scenario for Kalanchoe pinnata in an invaded seasonally dry tropical forest
Source: Ecol Evol. 2016 Jun 9;6(13):4541–50. doi: 10.1002/ece3.2219 (PMC4931000; doi:10.1002/ece3.2219)
Supplement: Supplementary file 2 — Table S1. Summary of vital rates estimation; P ij values were estimated for 1 year discrete transitions. Table S2. Summary of contrasts for viability and germination of seeds between pollination treatments applied in Kalanchoe pinnata based on 234 degrees of freedom. [file ECE3-6-4541-s002.docx]

Table S1. Summary of vital rates estimation; P_ij_ values were estimated for one year discrete transitions.

|  | Life cycle vital rate | Vital rate estimation |
| --- | --- | --- |
| S_11_ | Seed stasis in the seed bank | 15 viable seeds/937 one year old seeds |
| S_22_ | Sexual plantlet survival | 5 surviving plantlets / 84 initial plantlets |
| S_33_ | Asexual plantlet survival | 14 surviving plantlets / 114 initial plantlets |
| S_44_ | Juvenile survival | 98 surviving juveniles/ 425 tagged juveniles |
| S_55_ | Adult survival | 0 (Semelparous plant) |
| G_21_ | Seed from the seedbank that germinate | viable seeds*(1 -germinated seed proportion); (S_11_)(0.986) |
| G_42_ | Seedling growth to juvenile | 7 juveniles / 84 sexual plantlets |
| G_43_ | Asexual plantlets growth to juvenile | 89 juveniles / 114 asexual plantlets |
| G_54_ | Flowering juveniles | 66 adults / 255 juveniles |
| R_15_ | Seeds added to the seed bank per adlut | (17 fructifications per plant)*(881 seed per fruit)*(1-germinated seed proportion) |
| R_25_ | Seeds germinated the year they are produced | (22 flowers)(0.77 non aborted ratio)(881 seed per fruit)(germinated ratio), germinated ratio= 92/6701 |
| R_34_ | Asexual plantlets per juvenile | (7 leaves dropped per juvenile * 0.69 plantlet per leaf |
| R_35_ | Asexual plantlets per adult | 19 leaves dropped per adult * 0.76 plantlet per leaf |

Table S2. Summary of contrasts for viability and germination of seeds between pollination treatments applied in *Kalanchoe pinnata* based on 234 degrees of freedom.

|  | Seed viability |  |  |  | Seed germination | |  |  |
| --- | --- | --- | --- | --- | --- | --- | --- | --- |
|  | Estimate | Standard error | T | P | Estimate | Standard error | T | P |
| Overall |  |  |  |  |  |  |  |  |
| NP-CP | -1.76 | 1.3 | 1.35 | 0.177 | -1.27 | 0.8 | 1.58 | 0.11 |
| NP-AP | 0.87 | 1.3 | 0.67 | 0.502 | 0.21 | 0.8 | 0.26 | 0.79 |
| NP-Em | 4.23 | 1.3 | 3.25 | 0.0013* | 1.17 | 0.8 | 1.46 | 0.14 |
| CP-AP | 2.63 | 1.3 | 2.02 | 0.044* | 1.48 | 0.8 | 1.85 | 0.06 |
| CP -Em | 6 | 1.3 | 4.6 | <0.001* | 2.45 | 0.8 | 3.04 | 0.002* |
| AP-Em | 3.36 | 1.3 | 2.58 | 0.01* | 0.96 | 0.8 | 1.2 | 0.23 |
|  |  |  |  |  |  |  |  |  |
| 2013 |  |  |  |  |  |  |  |  |
| NP-CP | 1.324 | 3.98 | 0.33 | 0.74 | -2.026 | 2.458 | 0.82 | 0.41 |
| NP-AP | 2.87 | 3.98 | 0.72 | 0.47 | 3.065 | 2.458 | 1.24 | 0.21 |
| NP-Em | 4.64 | 3.91 | 1.18 | 0.23 | 4.928 | 2.418 | 2.03 | 0.042* |
| CP-AP | 1.54 | 3.51 | 0.44 | 0.66 | 5.09 | 2.168 | 2.34 | 0.019* |
| CP -Em | 3.31 | 3.44 | 0.96 | 0.33 | 6.954 | 2.122 | 3.27 | 0.001* |
| AP-Em | 1.77 | 3.44 | 0.515 | 0.6 | 1.863 | 2.122 | 0.87 | 0.38 |
|  |  |  |  |  |  |  |  |  |
| 2014 |  |  |  |  |  |  |  |  |
| NP-CP | -4.84 | 4.76 | 1.02 | 0.309 | -0.52 | 2.9 | 0.18 | 0.85 |
| NP-AP | -1.12 | 4.7 | 0.23 | 0.81 | -2.63 | 2.9 | 0.89 | 0.36 |
| NP-Em | 3.83 | 4.37 | 0.81 | 0.42 | -2.57 | 2.69 | 0.88 | 0.37 |
| CP-AP | 3.72 | 4.31 | 0.85 | 0.39 | -2.11 | 2.66 | 0.78 | 0.43 |
| CP -Em | 8.68 | 4.31 | 2.01 | 0.045* | -2.05 | 2.66 | 0.77 | 0.44 |
| AP-Em | 4.95 | 4.76 | 1.147 | 0.252 | 0.06 | 2.93 | 0.02 | 0.98 |

Figure S1. Management on simulated populations of *Kalanchoe pinnata*, when: elastic (a), sensible (b) or both (c) life traits are removed annually at a rate of 80%. White lines correspond to the mean trend and each polygon indicates the standard deviation of 1000 simulations.
